# Additional Minor Diterpene Glycosides from *Stevia rebaudiana* Bertoni

**DOI:** 10.3390/molecules181113510

**Published:** 2013-10-31

**Authors:** Indra Prakash, Venkata Sai Prakash Chaturvedula

**Affiliations:** Organic Chemistry Department, The Coca-Cola Company, Global Research and Development, One Coca-Cola Plaza, Atlanta, GA 30313, USA

**Keywords:** *Stevia rebaudiana*, Compositae, Asteraceae, diterpenoid glycosides, spectral data, chemical studies, structure characterization

## Abstract

Two additional novel minor diterpene glycosides were isolated from the commercial extract of the leaves of *Stevia rebaudiana* Bertoni. The structures of the new compounds were identified as 13-{β-D-glucopyranosyl-(1→2)-*O*-[β-D-glucopyranosyl-(1→3)-β-D-glucopyranosyl-oxy} *ent*-kaur-16-en-19-oic acid {β-D-xylopyranosyl-(1→2)-*O*-[β-D-glucopyranosyl-(1→3)]-*O*-β-D-glucupyranosyl-ester} (**1**), and 13-{β-D-6-deoxy-glucopyranosyl-(1→2)-*O*-[β-D-glucopyranosyl-(1→3)-β-D-glucopyranosyl-oxy} *ent*-kaur-16-en-19-oic acid {β-D-glucopyranosyl-(1→2)-*O*-[β-D-glucopyranosyl-(1→3)-β-D-gluco-pyranosyl-ester} (**2**), on the basis of extensive 1D (^1^H- and ^13^C-) 2D NMR (COSY, HSQC and HMBC) and MS spectroscopic data as well as chemical studies.

## 1. Introduction

The major constituents isolated from the leaves of a perennial shrub *Stevia rebaudiana* Bertoni (family: Asteraceae) are the potently sweet diterpenoid glycosides stevioside and rebaudioside A. Extracts of the leaves of *S. rebaudiana* native to Brazil and Paraguay have been used for centuries to sweeten food and beverages in Japan, South America and China. Due to the increase in demand for the major constituents in the leaves of *S. rebaudiana* which are the potently sweet diterpenoid glycosides stevioside, rebaudiosides A and D, and dulcoside A, it is now grown commercially in a number of countries, particularly in China, Japan, Taiwan, Korea, Thailand and Indonesia [1,2]. The compounds isolated from *S. rebaudiana* known as stevia sweeteners are the glycosides of the diterpene steviol, *ent*-13-hydroxykaur-16-en-19-oic acid [3].

In our continuing research to discover novel natural sweeteners, we have isolated several new steviol glycosides from the commercial extracts of the leaves of *S. rebaudiana* [4,5,6,7]. Apart from isolation and structural characterization of novel compounds from *S. rebaudiana*, and their possible utilization as natural sweeteners or sweetness enhancers, we have also studied the stability of many steviol glycosides in various systems of interest and characterized their degradation products using a number of spectroscopic methods [8]. We are also engaged in the synthesis of selected steviol diterpene glycosides using naturally occurring starting materials [9,10,11].

## 2. Results and Discussion

**Figure 1:**
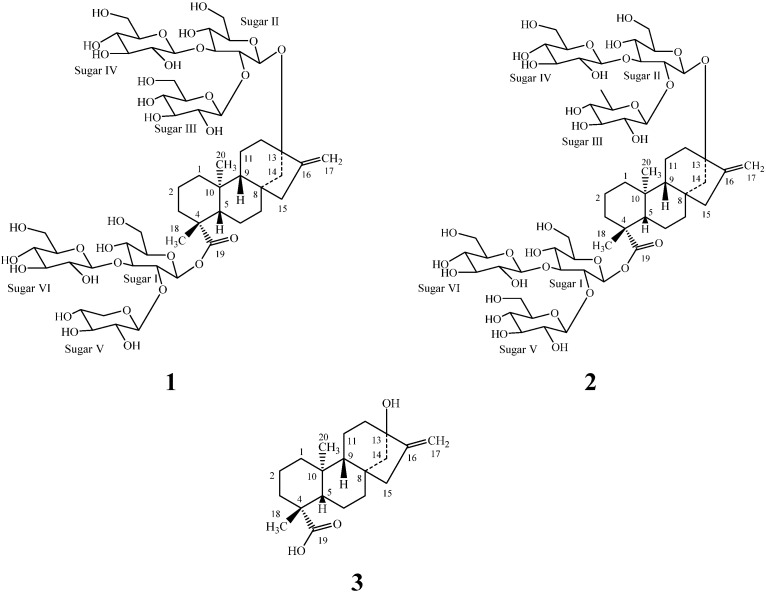
Structures of **1**, **2** and steviol (**3**).

Purification of the commercial extract of rebaudioside M (also known as rebaudioside X) obtained from the leaves of *S. rebaudiana* from Lot No: PT01021 of Pure Circle, Malaysia resulted in the isolation of the two new diterpenoid glycosides 13-{β-D-glucopyranosyl-(1→2)-*O*-[β-D-glucopyranosyl-(1→3)-β-D-glucopyranosyl-oxy} *ent*-kaur-16-en-19-oic acid {β-D-xylopyranosyl-(1→2)*-O-*[β-D-gluco-pyranosyl-(1→3)]*-O-*β-D-glucupyranosyl-ester} (**1**), and 13-{β-D-6-deoxy-glucopyranosyl-(1→2)*-O-*[β-D-glucopyranosyl-(1→3)-β-D-glucopyranosyl-oxy} *ent*-kaur-16-en-19-oic acid {β-D-glucopyranosyl-(1→2)*-O-*[β-D-glucopyranosyl-(1→3)-β-D-glucopyranosyl-ester} (**2**) (Figure 1).

Compound **1** was obtained as a white powder and its molecular formula was deduced as C_55_H_88_O_32_ from its HRESI mass spectrum in the positive mode which showed a (M+H)^+^ ion at *m/z* 1261.5353; this was supported by the ^13^C-NMR spectral data. The ^1^H-NMR spectrum of **1** showed the presence of two methyl signals resonating at δ 1.29 and 1.36 as singlets, two olefinic proton singlets at δ 4.89 and 5.69 corresponding to an exocyclic double bond, nine methylene and two methine protons between δ 0.75–2.70, characteristic for the *ent*-kaurane diterpenoids isolated earlier from the genus *Stevia* [6,7,8,9]. The basic skeleton of *ent-*kaurane diterpenoids was supported by the key COSY: H-1/H-2; H-2/H-3; H-5/H-6; H-6/H-7; H-9/H-11; H-11/H-12, and HMBC correlations: H-1/C-2, C-10; H-3/C-2, C-4, C-5, C-18, C-19; H-5/C-4, C-6, C-10, C-18, C-19, C-20; H-9/C-8, C-10, C-11; H-14/C-8, C-9, C-13, C-15, C-16 and H-17/C-13, C-15, C-16) correlations [4,5,6,7,8,9,10,11]. The presence of six sugar units in its structure was evident by the presence of the anomeric protons resonating at δ 5.33, 5.45, 5.46, 5.48, 5.62, and 6.38 in its ^1^H-NMR spectrum. The presence of these six sugar units was further supported by the MS/MS spectrum of **1** in the positive ESI mode that showed fragment ions at *m/z* 1129, 967, 805, 643, 481 and 319; suggesting the presence of a pentose and five hexose moieties in its structure. Acid hydrolysis of **1** with 5% H_2_SO_4_ afforded the sugars D-glucose and D-xylose, which were identified by direct comparison with authentic samples by TLC [12,13,14]. The anomeric proton observed at δ 6.38 showed an HMBC correlation to C-19 which indicated that it corresponds to the anomeric proton of Sugar I. Similarly, the anomeric proton observed at δ 5.45 showed an HMBC correlation to C-13 allowing it to be assigned as the anomeric proton of Sugar II. The sequence in sugar units and the assignments for C-2 through C-6 of Glc_V_ and Glc_VI_ were made using the ^1^H-NMR, COSY and HSQC data. Further, the identification of sugars present in **1** and their configurations were achieved by preparing their thiocarbamoyl-thiazolidine carboxylate derivatives with L-cysteine methyl ester and *O*-tolyl isothiocyanate, and comparison of their retention times with the standard sugars as described in the literature; suggesting the sugar moieties present as β-D-glucopyranosyl and β-D-xylopyranosyl units [15]. Enzymatic hydrolysis of **1** furnished an aglycone which was identified as steviol (**3**) by comparison of ^1^H-NMR and co-TLC with a standard [16]. The large coupling constants observed for the six anomeric protons of the sugar moieties at δ 5.33 (d, *J* = 8.1 Hz), 5.45 (d, *J* = 7.8 Hz), 5.46 (d, *J* = 7.5 Hz), 5.48 (d, *J* = 7.9 Hz), 5.62 (d, *J* = 7.8 Hz), and 6.38 (d, *J* = 8.4 Hz), suggested their β-orientation, as reported for steviol glycosides [4,5,6,7,8,9,10,11]. The ^1^H- and ^13^C-NMR values for all the proton and carbons were assigned on the basis of 2D NMR spectral data (COSY, HSQC and HMBC) and are given in Table 1.

**Figure 2:**
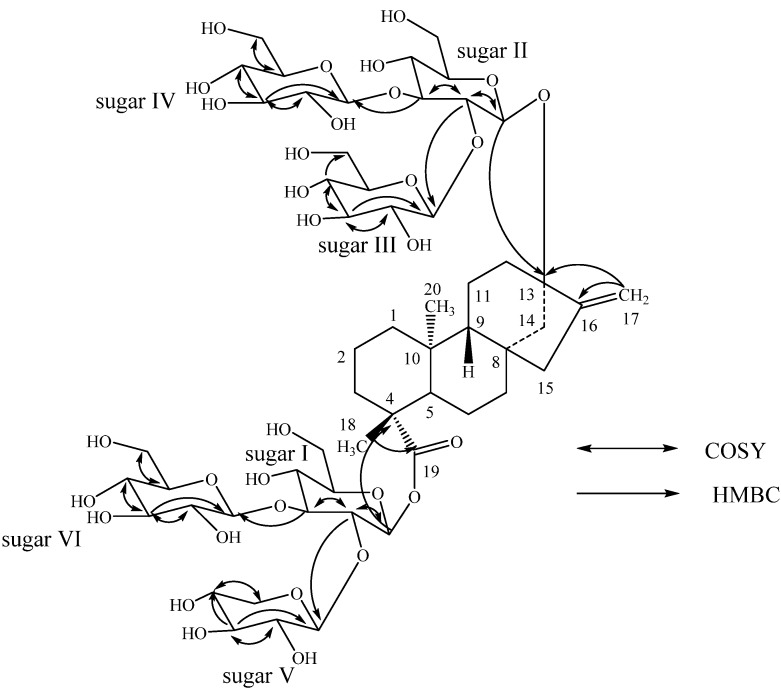
Key COSY and HMBC correlations of **1**.

From the above NMR spectral data and hydrolysis studies, it was inferred that there are five β-D-glucopyranosyl units and a β-D-xylopyranosyl unit in the structure of **1** connected to the aglycone steviol. Comparison of the ^1^H- and ^13^C-NMR values of **1** with rebaudioside D [4,17] suggested the presence of a steviol aglycone moiety with a 2,3-branched β-D-glucotriosyl substituent and another β-D-glucosyl moiety in the form of an ester at C-19, leaving the assignment of the additional β-D-glucosyl and β-D-xylosyl moieties. The downfield shift for both the ^1^H and ^13^C chemical shifts at C-2' and C-3' of sugar I (C-19 position) suggested that the additional β-D-xylosyl and β-D-glucosyl moieties are attached at thse positions respectively. This was confirmed by the key HMBC correlations as shown in Figure 2. Based on the results from chemical and spectral studies, **1** was assigned as 13-{β-D-glucopyranosyl-(1→2)*-O-*[β-D-glucopyranosyl-(1→3)-β-D-glucopyranosyl-oxy} *ent*-kaur-16-en-19-oic acid {β-D-xylopyranosyl-(1→2)*-O-*[β-D-glucopyranosyl-(1→3)]*-O-*β-D-glucupyranosyl-ester}.

**Table 1:** ^1^H- and ^13^C-NMR chemical shift values for **1**–**2** isolated from *Stevia rebaudiana* Bertoni recorded in d5-pyridine (C_5_D_5_N) ^a–c^.

Position	1		2	
	^1^H-NMR	^13^C-NMR	^1^H-NMR	^13^C-NMR
1	0.75 br t (13.2), 1.76 m	40.0	0.77 br t (12.3), 1.79 m	39.9
2	1.35 m, 2.23 m	19.4	1.37 m, 2.28 m	19.3
3	1.01 m, 2.29 m	38.0	1.02 m, 2.29 m	38.1
4	−	43.9	−	44.0
5	1.02 br d (13.0)	57.0	1.06 br d (12.9)	57.0
6	2.07 m, 2.42 q (13.5)	23.1	2.24 m, 2.41 m	23.2
7	1.37 m, 1.73 m	42.2	1.42 m, 1.81 m	42.3
8	−	39.2	−	39.7
9	0.90 br d (8.1)	54.0	0.92 br d (7.7)	54.0
10	−	41.3	−	41.4
11	1.65 m, 1.75 m	19.9	1.67 m, 1.76 m	19.8
12	1.86 m, 2.70 m	38.2	1.81 m, 2.74 m	38.1
13	−	87.5	−	87.4
14	2.01 m, 2.72 m	42.9	2.01 m, 2.75 m	42.9
15	1.88 d (17.0), 2.04 m	46.3	1.88 m, 2.05 m	46.1
16	−	153.9	−	153.7
17	4.89 s, 5.69 s	104.6	4.89 s, 5.72 s	104.5
18	1.29 s	27.9	1.32 s	27.9
19	−	176.7	−	177.0
20	1.36 s	16.4	1.38 s	16.6
1'	6.38 d (8.4)	94.4	6.41 d (8.2)	94.5
2'	4.38 m	77.1	4.52 t (8.7)	76.5
3'	5.04 m	88.2	5.14 t (8.7)	88.2
4'	4.24 m	69.8	4.20 m	69.7
5'	4.14 m	78.3	4.14 m	78.0
6'	4.20 m 4.33 m	61.7	4.20 m, 4.30 m	61.7
1''	5.45 d (7.8)	96.0	5.49 d (7.9)	95.9
2''	4.13 m	81.0	4.08 m	81.1
3''	4.98 t (9.1)	87.6	5.01 m	87.7
4''	4.08 t (9.1)	70.1	4.09 m	70.3
5''	3.95 m	77.4	3.96 m	77.4
6''	4.21 m, 4.35 m	62.4	4.20 m, 4.32 m	62.2
1'''	5.48 d (7.9)	104.5	5.39 d (8.0)	104.3
2'''	4.16 m	75.3	4.14 m	75.5
3'''	4.13 m	78.2	4.04 m	78.1
4'''	3.99 m	72.9	3.69 t (8.9)	76.7
5'''	3.75 ddd (3.1, 6.5, 9.7)	77.3	3.47 dq (6.0, 8.9)	72.4
6'''	4.28 m, 4.51 dd (1.1, 11.6)	63.6	1.63 d (6.1)	18.3
1''''	5.46 d (7.5)	103.8	5.48 d (7.9)	103.5
2''''	3.98 m	75.2	4.00 m	75.2
3''''	4.47 t (8.6)	77.6	4.55 t (9.2)	77.4
4''''	4.14 m	70.9	4.18 m	71.0
5''''	3.99 m	77.7	4.01 m	77.6
6''''	4.20 m, 4.33 m	61.7	4.20 m, 4.30 m	61.7
1'''''	5.62 d (7.8)	104.7	5.81 d (7.5)	103.9
2'''''	4.17 m	75.2	4.25 m	75.0
3'''''	4.12 m	78.3	4.18 m	78.0
4'''''	4.32 m	71.3	4.10 m	73.3
5'''''	3.54 t (11.0), 4.32 m	66.6	3.90 ddd (3.3, 6.8, 9.3)	77.4
6'''''	−	−	4.32 m, 4.66 d (11.3)	63.6
1''''''	5.33 d (8.1)	103.9	5.30 d (8.0)	103.9
2''''''	3.97 m	75.2	3.95 m	75.1
3''''''	4.38 m	77.5	4.33 m	77.6
4''''''	4.11 m	70.9	4.09 m	70.6
5''''''	3.87 m	77.8	3.82 m	77.7
6''''''	4.10 m, 4.31 m	61.8	4.10 m, 4.32 m	61.7

^a^ assignments made on the basis of COSY, HSQC and HMBC correlations; ^b^ Chemical shift values are in δ (ppm); ^c^ Coupling constants are in Hz.

The molecular formula of compound **2** was determined to be C_56_H_90_O_32_ by the [M+H]^+^ ion at *m/z* 1,275.5485 in the positive ESI mass spectrum which was further supported by the ^13^C-NMR spectral data. The ^1^H-NMR spectrum of **2** also showed the presence of two methyl singlets, nine methylene and two methine protons as in 1. The positive ESI MS/MS spectrum of **2** showed the fragment ions at *m/z* 1,129, 967, 805, 643, 481, and 319 corresponding to the successive loss of a deoxyhexose and five hexose units from its [M+H]^+^ ion, which was supported by the six anomeric protons observed at δ 5.30, 5.39, 5.48, 5.49, 5.81, 6.41 in its ^1^H-NMR spectral data. Acid hydrolysis of **2** performed using the same procedure described as in **1** afforded D-glucose and D-deoxyglucose confirming the presence of a D-deoxypyranosyl and five D-glucopyranosyl units in its molecular structure. The two sugars present in **2** were identified as β-D-glucopyranosyl and β-D-6-deoxygluco-pyranosyl units by preparing their thiocarbamoyl-thiazolidine carboxylate derivatives with L-cysteine methyl ester and *O*-tolyl isothiocyanate, as in **1**. The large coupling constants observed for the six anomeric protons suggested their β-orientation, as reported for steviol glycosides such as in the case of **1**.

**Figure 3:**
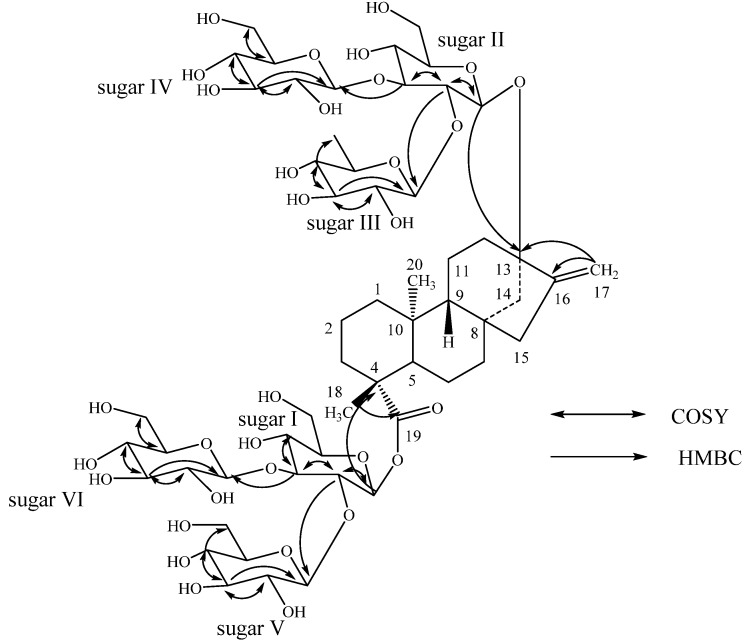
Key COSY and HMBC correlation of **2**.

Enzymatic hydrolysis of **2** furnished a compound which was found identical to steviol (**3**) as in **1**. A close comparison of the ^1^H- and ^13^C-NMR values of **2** with **1** and rebaudioside D suggested the presence of three glucose units attached as a 2,3-branched β-D-glucotriosyl substituent at C-19 carboxylic acid with another β-D-glucosyl moiety at C-13 hydroxyl leaving the assignment of the additional β-D-glucosyl and a β-D-deoxyglucosyl moieties. From the key COSY and HMBC correlations shown in Figure 3, it was concluded that β-D-deoxyglucosyl and β-D-glucosyl units have been connected at C-2 and C-3 positions respectively to the β-D-glucosyl moiety at C-13 position. Thus, the structure of the novel compound **2** has been deduced as 13-{β-D-6-deoxy-glucopyranosyl-(1→2)*-O-*[β-D-glucopyranosyl-(1→3)-β-D-glucopyranosyl-oxy} *ent*-kaur-16-en-19-oic acid {β-D-glucopyranosyl-(1→2)*-O-*[β-D-glucopyranosyl-(1→3)-β-D-glucopyranosyl-ester}.

## 3. Experimental

### 3.1. General

NMR spectra were acquired on a Bruker Avance DRX 500 MHz instrument with a 5 mm inverse detection probe using standard pulse sequences. The spectrum was referenced to the residual solvent signal (δ_H_ 8.71, δ_C_ 149.9 for pyridine-*d*_5_), chemical shifts are given in δ (ppm), and coupling constants are reported in Hz. High Resolution Mass Spectral (HRMS) and MS/MS fragmentation pattern data were generated in the positive-ion mode using a mass spectrometer (Waters Premier Quadrupole Time-of-Flight) equipped with an electrospray ionization source. Samples were diluted with water: acetonitrile (1:1) containing 0.1% formic acid and introduced via infusion using the onboard syringe pump.

### 3.2. Plant Material

The commercial rebaudioside M with Lot No: PT01021 was obtained from Pure Circle, Malaysia. A voucher specimen is deposited at The Coca-Cola Company, No. VSPC-2973-6B.

### 3.3. Isolation

**Figure 4:**
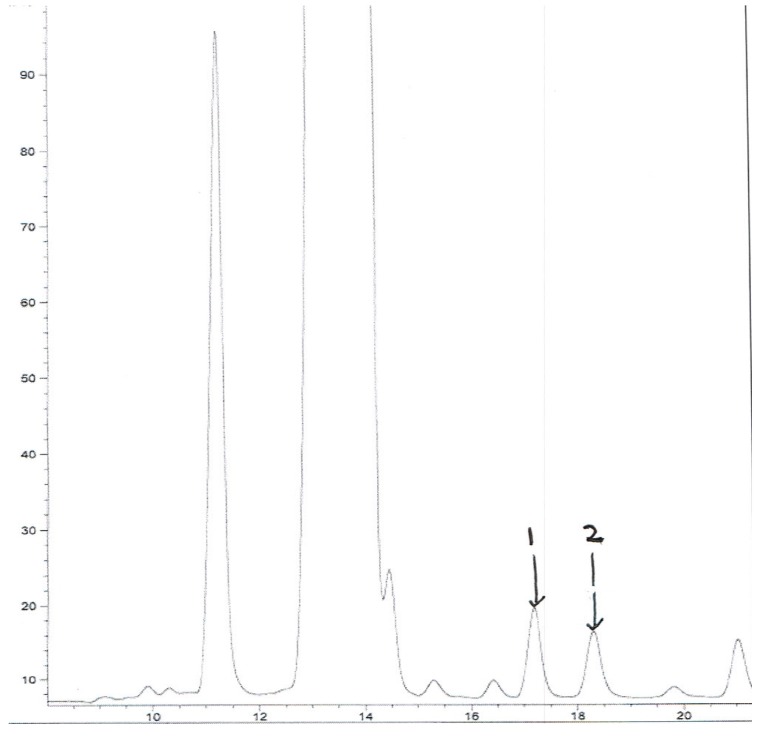
HPLC Trace of mixture of **1** and **2**.

Isolation of the pure compounds **1**–**2** has been carried out using an Agilent 1100 HPLC fractionation of a sample of rebaudioside M (VSPC-2973-6B, 2 g). The HPLC method involved using Phenomenex Prodigy Column C_18_ (250 × 10 mm, 10 μm); UV Detection: 210 nm; Mobile Phase A: H_2_O (0.1% TFA); Mobile Phase B: acetonitrile; Flow Rate: 20.0 mL/min; Injection volume: 1,400 μL. The gradient increased from 75:25 (A:B) to 69:31 (A:B) over 20 min, and remained at 50:50 (A:B) for 5 min. The peaks eluting at *t_R_* 17.18 and 18.29 min were collected over several injections and concentration under vacuum furnished **1** (2.4 mg) and **2** (1.8 mg) respectively (Figure 4).

*13-{β-D-Glucopyranosyl-(1→2)-O-[β-D-glucopyranosyl-(1→3)-β-D-glucopyranosyl-oxy} ent-kaur-16-en-19-oic acid {β-D-xylopyranosyl-(1→2)-O-[β-D-glucopyranosyl-(1→3)]-O-β-D-glucupyranosyl-ester}* (**1**); White powder, ^1^H-NMR (500 MHz, C_5_D_5_N, δ ppm) and ^13^C-NMR (125 MHz, C_5_D_5_N, δ ppm) spectroscopic data see Table 1; HRESIMS (M+H)^+^ *m/z* 1261.5353 (calcd. for C_55_H_89_O_32_: 1261.5337).

*13-{β-D-6-Deoxy-glucopyranosyl-(1→2)-O-[β-D-glucopyranosyl-(1→3)-β-D-glucopyranosyloxy} ent-kaur-16-en-19-oic acid {β-D-glucopyranosyl-(1→2)-O-[β-D-glucopyranosyl-(1→3)-β-D-gluco-pyranosyl-ester}* (**2**); White powder, ^1^H-NMR (500 MHz, C_5_D_5_N, δ ppm) and ^13^C-NMR (125 MHz, C_5_D_5_N, δ ppm) spectroscopic data see Table 1; HRESIMS (M+H)^+^ *m/z* 1275.5485 (calcd. for C_56_H_91_O_32_: 1275.5493).

*Acid hydrolysis of* **1** *and* **2**. To a solution of compounds **1** and **2** (500 μg) in MeOH (1 mL) was added 5% H_2_SO_4_ (0.5 mL) and the mixture was refluxed for 24 h. The resulting reaction mixture was extracted with ethyl acetate (EtOAc, 2 × 10 mL) after neutralization with saturated aqueous sodium carbonate solution yields an aqueous fraction containing sugars. The sugars present in the aqueous phase were identified as D-glucose and D-xylose in **1**, and D-glucose and 6-deoxy-D-glucose in **2**, by comparing with standard sugars using the TLC systems EtOAc/*n*-butanol/water (2:7:1) and CH_2_Cl_2_/MeOH/water (10:6:1) [12,13,14].

*Determination of sugar configuration in* **1*** and* **2***.* Each compound **1**–**2** (1 mg) was hydrolyzed with 0.5 M HCl (2 mL) for 1.5 h. The reaction mixture was cooled to room temperature, passed through an Amberlite IRA400 column and the eluate was lyophilized. The residue was dissolved in pyridine (1 mL) and heated with L-cysteine methyl ester HCl (5 mg) at 60 °C for 1.5 h, and then *O*-tolyl isothiocyanate (25 μL) was added to the mixture and heated at 60 °C for an additional 1.5 h. The reaction mixture was analyzed by HPLC: column Phenomenex Luna C_18_, 150 × 4.6 mm (5 µL); Mobile phase: 25% acetonitrile in water with 0.2% TFA, Flow rate: 1 mL/min; UV detection at 250 nm. The sugars were identified as D-glucose (*t_R_*, 12.34 min) and D-xylose (*t_R_*, 14.08) in **1**, whereas D-glucose (*t_R_*, 12.26 min) 6-deoxy-D-gluose (*t_R_*, 11.34 min) in **2** [(authentic samples, D-glucose (*t_R_*, 12.64) and L-glucose (*t_R_*, 11.36 min); D-xylose (*t_R_*, 14.23) and L-xylose (*t_R_*, 13.06 min); 6-deoxy-D-glucose (*t_R_*, 11.04) and 6-deoxy-L-glucose (*t_R_*, 17.68)] [15].

*Enzymatic hydrolysis of* **1*** and* **2**. To a solution of each compound **1**–**2** (250 μg) was added 0.1 M sodium acetate buffer, pH 4.5 (5 mL) and crude pectinase from *Aspergillus niger* (P2736, 50 μL, Sigma-Aldrich, St. Louis, MO, USA). The mixture was stirred at 50 °C for 120 h. The product precipitated out during the reaction and was filtered and then crystallized from methanol (MeOH). The resulting product was identical to *ent*-13-hydroxykaur-16-en-19-oic acid (steviol, **3**) by comparison of their ^1^H-NMR spectral data and co-TLC [16].

## 4. Conclusions

Two novel diterpenoid glycosides **1**–**2** were isolated from a commercial sample of rebaudioside M of the leaves of *S. rebaudiana* Bertoni obtained from Pure Circle, Malaysia. The structure of the new compounds were identified as 13-{β-D-glucopyranosyl-(1→2)*-O-*[β-D-glucopyranosyl-(1→3)-β-D-glucopyranosyl-oxy} *ent*-kaur-16-en-19-oic acid {β-D-xylopyranosyl-(1→2)*-O-*[β-D-glucopyranosyl-(1→3)]*-O-*β-D-glucupyranosyl-ester} (**1**), and 13-{β-D-6-deoxyglucopyranosyl-(1→2)*-O-*[β-D-gluco-pyranosyl-(1→3)-β-D-glucopyranosyl-oxy} *ent*-kaur-16-en-19-oic acid {β-D-glucopyranosyl-(1→2)*-O-*[β-D-glucopyranosyl-(1→3)-β-D-glucopyranosyl-ester} (**2**), on the basis of spectroscopic and chemical studies. The discovery of these two novel compounds is an important addition in expanding our understanding of the diversity of the diterpenoid glycosides present in the *S. rebaudiana* Bertoni. This is the first report of the isolation of the two novel diterpene glycosides **1**–**2** from *S. rebaudiana* Bertoni and their complete ^1^H- and ^13^C-NMR spectral assignments that were made on the basis of spectral (COSY, HSQC, HMBC, and MS/MS) and chemical studies.
